# Evaluation of Antibacterial Effect against* Porphyromonas gingivalis* and Biocompatibility of Essential Oil Extracted from the Gum of* Pistacia atlantica Kurdica*

**DOI:** 10.1155/2019/9195361

**Published:** 2019-05-29

**Authors:** Shokhan H. Azeez, Shanaz M. Gaphor

**Affiliations:** ^1^Department of Dental Nursing, Sulaimani Technical Institute, Sulaimani Polytechnic University, Sulaimani, Kurdistan, Iraq; ^2^Department of Oral Diagnosis, College of Dentistry/University of Sulaimani, Sulaimani, Kurdistan, Iraq

## Abstract

**Objective:**

This study investigates the antibacterial effect of essential oil extracted from the gum of* Pistacia atlantica Kurdica* against* Porphyromonas gingivalis* and histological evaluation of an experimental gel of the extract in wound healing in the back subcutaneous tissue of Wistar rats.

**Methods:**

Clinical strains of bacteria obtained from subgingival plaque samples of individuals having periodontitis, isolation, and confirmation were done by conventional microbiological tests and molecular technique. Essential oil was extracted by using hydrodistillation method; antibacterial activity has been determined by two fold serial dilution method. Histological evaluation conducted on fifteen Wistar rats. Incisions were made on the dorsal surface of each animal for implanting of 3 polysilicone tubes (empty, tetracycline gel, and the experimental gel). After 1, 4, and 8 weeks, the animals were euthanized and the specimens were prepared histologically.

**Result:**

The extract demonstrated antimicrobial effect and significant wound healing in the different study durations particularly our product showed progression in epidermal wound healing and decrease in cellularity and scoring of inflammatory cells.

**Conclusion:**

The extract was able to pose inhibitory and bactericidal activity against* P. gingivalis* and the experimental gel was able to have a good wound healing that enable it to be considered as a compatible material.

## 1. Introduction

Pistacia is a genus of Anacardiaceae family, of about 11 or more species, which are shrubs or trees. Three Pistacia species (*P. vera Linnaeus*,* P. khinjuk Stocks*, and* P. Atlantica)* naturally occur,* Pistacia atlantica Kurdica *is the wildest species widely distributed, from southern-west Asia to northern-west Africa [1]. A large number of wild* Pistacia atlantica *subsp.* Kurdica* trees are widely distributed in the Zagros Mountains; West and North of Iran, East and North of Iraq, South of Turkey, and North of Syria [2]. The mastic gum exudates from the plant stem traditionally have been used in Greek medicine for treatment of gastrointestinal disorders like gastralgia, dyspepsia, and peptic ulcer for many years [3]; the gum possess considerable antibacterial and antifungal activity [4]. Essential oils are fragrant volatile substances biosynthesized by several parts of plants, which is commonly used for many years for combating pathogens such as bacteria, fungi, and viruses [5]. The terpenoids and some hydrocarbons that found in essential oils are responsible for their antimicrobial effect [6]. Studies suggested that terpenoids that diffuse within the cell membrane of bacteria, damaging it irreversibly, lead to bacterial death, because they are lipophilic in nature; these terpenes cause expansion of the membrane, increase fluid flow, and deactivate the enzymes placed within the membrane [7]. Pistacia species have caught the interest of researchers due to studies on different parts of this plant such as the leaves, kernels, hulls, and gum demonstrating various biological benefits such as antioxidant, antimicrobial, anti-inflammatory potential [8]. It has been proven that Pistachio species are a rich source of phenolic compounds and they have also been considered a source of high antioxidant activity [9].

*Porphyromonas gingivalis* is major periodontal pathogens and one of the main etiological agents in the inflammatory episodes of periodontal disease initiation and progression [10]. Plant-derived essential oil products have been widely used as a therapeutic agent in the treatment of periodontitis [11].

Biocompatibility (or tissue compatibility) is the capacity of a material to promote adequate host response when introduced as purposed [12]. Several organizations, such as the Food and Drug Administration, American National Standards Institute, International Organization for Standardization (ISO), and certain accessory bodies such as the Nordic Institute of Dental Materials and the European Union, are working diligently on the materials to be tested [13]

Tetracyclines are a group of antibiotics that possess anti-inflammatory, antiapoptotic, antiproteolytic, and antiangiogenic effects [14]. The chemically modified tetracyclines (CMTs) have the nonantimicrobial characteristics of the original compounds, such as regulation of release of inflammatory cytokines, activation or inhibition of leukocyte chemotaxis action, and antioxidative properties [15].

The data concerning the efficacy of this extracted oil against periopathogens is limited. The objective of this work is to determine the antibacterial activity of the extracted essential oil from* Pistacia atlantica Kurdica *gum against the growth of clinical isolates of* Porphyromonas gingivalis* and evaluate a histopathological examination of experimental gel of essential oil of* Pistacia atlantica Kurdica *gum in the back subcutaneous tissue of Wistar rats. The tissue inflammatory reaction surrounding the opening of the experimental gel was compared with an empty tube (control negative) and tetracycline gel (control positive) was used to determine biocompatibility of the tested material.

## 2. Materials and Methods

### 2.1. Plant Material and Essential Oil Extraction

The gum of* Pistacia atlantica Kurdica (*pistachio tree of the Atlas) was collected in June-July 2017 from the Halabja region, 70 km from Sulaimani city in the north of Iraq. The essential oil was extracted from the gum by hydrodistillation: 100g of gum was soaked in 350 ml of distilled water in a conical flask and left for 3 hours using a Clevenger apparatus according to published procedure [16, 17]. Finally, the essential oil was collected after decantation.

### 2.2. Gas Chromatography-Mass Spectrometry (GC-MS) Analysis

To identify and analyze the constituents of the essential oil of* Pistacia atlantica Kurdica* gum, it was analyzed by (GCMS-QP2010 Ultra) GC systems coupled to a mass spectrometer.

### 2.3. Plaque Sampling

This study involves clinical strain of* P. gingivalis *Gram-negative bacteria isolated from periodontal pocket of patients attended Shorsh dental center in Sulaymaniya city/Iraq in August 2017, subgingival plaque samples were collected from systematically healthy persons suffering from chronic periodontitis, from periodontal pockets with of at least six mm depth with sterilized paper point, then the samples were spread on Colombia agar media (LAB001 UK) supplemented with sheep blood %5, 5*μ*g/ml hemin (sigma Aldrich-china), 1*μ*g/ml vitamin K1 (Himedia), 7.68 mg colistin methanesulfonate (Himedia), 5 mg bacitracin (Himedia), and 7.5 mg nalidixic acid (Himedia) according to [18]. Plates were incubated for 7-10 days anaerobically using an anaerobic jar and anaerobic gas packs (AnaeroGen® system Oxoid), patients' consent and approvals were obtained prior to collecting the samples.

### 2.4. Identification and Isolation of the Microorganism

The clinical strain of Porphyromonas gingivalis was identified according to [18] by the morphological characteristic, Gram stain, Biochemical tests (VITEC 2), and PCR.

### 2.5. DNA Isolation

A colony was taken from bacterial cultures and mixed with 50*μ*L of sterilized deionized distill water (ddH2O) in microcentrifuge tube and then vortexed well until homogenized and incubated in a heat block for 10 minutes in 95°C. Finally, samples were spined down and the supernatant (DNA) was used as a template.

#### 2.5.1. DNA Amplification

A specific primer pairs for* P. gingivalis* on 16s rRNA was used for DNA amplification according to [19] which was published as follows which was at 72°C.

  Forward primer: 5′-AGGCAGCTTGCCATACTGCG – 3
  Reverse primer: 5′- ACTGTTAGCAACTACCGATGT - 3′

#### 2.5.2. PCR Product Preparation

A final volume of 20*μ*L containing 2*μ*L of reverse primer (10 pmol/*μ*L) (GeNet BioG-2000), 2*μ*L of forward primer (10 pmol/*μ*L) (GeNet BioG-2000), 10 *μ*L of 2X Prime Taq Premix (GeNet BioG-2000) which contain Prime Taq DNA Polymerase, reaction buffer, enzyme stabilizer, dNTPs mixture, and loading dye, 1*μ*L of ddH2O, and 5 *μ*L of DNA template was mixed.

#### 2.5.3. PCR Protocol

Consisted of initial denaturation for 5 minutes at 95°C for 1 cycle followed by amplification step which was repeated for 35 cycle that consisted of denaturation of DNA template at 95°C for 30 seconds; annealing of specific primers (for annealing temperature see Table 1) for 30 sec, and extension of primers at 72°C for 30 sec., the final extension for 5 min in 72°C for 1 cycle in Thermo cycler PCR (Verity TM 96 well, Applied Biosystem-USA) was done.

PCR product was analyzed by 2% agarose gel electrophoresis at 80 V for 35 min. Gel was stained with 3*μ*L of ethidium bromide and photographed on gel cabinet (Cleaver scientific Ltd., UK); 100bp plus DNA marker (cat no. M 2000) was used as a molecular weight marker. The gel purification was done for the bands by using the gene JET™ Gel extraction kit (# K0691) from Fermentas UK. Finally, standard sequencing for the PCR products was done by (Macrogen-South Korea).

### 2.6. The Sensitivity of P. gingivalis to Different Concentrations of Essential Oil of Pistacia atlantica Kurdica Gum

The stock solution of the tested agent was prepared (800*μ*L/1 ml) by dissolving the essential oil in DMSO (dimethyl sulfate). For MIC, twelve dilutions of the extract were prepared by using Muller Hinton broth medium-twofold serial dilution method. For dilutions, 1 ml from the stock solution was added to the first tube which contains 1 ml of Muller Hinton broth. Then 1 ml of Muller Hinton broth from the first tube was transferred into the second tube. The serial dilution was repeated for the tested agent till reaching 0.2*μ*ml/ml; thus the concentrations of serial dilution was 400, 200, 100, 50, 25, 12.5, 6.25, 3.12, 1.6, 0.8, 0.4, and 0.2.*μ*L/ml, respectively.

To each of the above 12 prepared MIC tubes with varying concentrations, 100*μ*L of bacterial suspension (5*∗*10 CFU/ml) prepared earlier was added to the tubes; thus the final volume was 1100*μ*L per tube. Tubes were sealed with cotton and incubated for ≥48 h at 37°C in an anaerobic jar by using AnaeroGen® system Oxoid gas pack and observed for turbidity. The minimum concentration of the essential oil in the tube which does not show any turbidity is considered as the MIC of the extract. Turbidity of the MIC tube indicated growth of the bacteria expressed that the bacteria are resistant to a tested agent.

### 2.7. Minimum Bactericidal Concentration

The determination of minimum bactericidal concentration was by choosing the concentrations that showed no bacterial growth during the evaluation of the MIC. For this, a sample was taken from the content of the chosen tubes by micropipette then spread on a blood agar plate using a sterile spreader and anaerobically incubated. After 48 h at 37°C, the plates were brought out and examined for bacterial growth; the plates that showed no growth of bacteria were identified as minimum bactericidal concentration. The experiments were repeated at least three times at each concentration and the modal values of MIC and MBC were selected.

### 2.8. Experimental Animals and Design

Fifteen male Wistar rats (randomly divided into three groups) weighing 200-250g were used. These animals were obtained from the College of Veterinary Medicine, University of Sulaimani; they were housed under a 12/12 h light/dark cycle with a temperature of 21 +/- 2°C and provided with water and food ad libitum. The handling of the animals was carried out according to the institutional guidelines and all experiments were performed in accordance with the Ethical Committee for Animal Research of Sulaimani University.

Disposable syringes of (22) gauge were used to deliver the experimental material inside sterile polysilicone tubes of 2 mm internal diameter and 10 mm length. In the control negative (CN; n=15) group the polysilicone tubes were left empty; in the control positive (PC; n=15) group the polysilicone tubes were filled with tetracycline gel; and in the experimental group (EG; n=15) the polysilicone tubes were filled with experimental gel prepared in the minimum inhibitory dose against* p. gingivalis* (12.5 *μ*l/L of essential oil of* Pistacia atlantica Kurdica *gum.

The rats in each group were subdivided into 3 subgroups according to the duration of the study periods (1, 4, and 8 days, with 5 rats in each group)

### 2.9. Treatment

The animals were anesthetized with a combination of a mixture of ketamine hydrochloride (3.3ml) and xylazine hydrochloride (2ml) by intraperitoneal injection at a dose of 0.05 mL/Kg/BW. The backs of the rats were shaved and disinfected with 0.12% chlorhexidine (Periogard; Pfizer Ltda, Santo Amaro, SP, Brazil). Four incisions were made in the dorsal surface of the rats (2 in the upper and 2 in the lower regions of the dorsum) using a sterile blade. The cutaneous tissues laterals to the incisions were twitched and dissection of the tissue was done using blunt-end scissors. Three tubes were applied or implanted in the back subcutaneous tissue of each animal: two tubes in the upper region (CN on the right of the incision and EG on the left) and one tube in the lower region (CP ). The borders of the incisions were sutured with nylon 5-0 (Ethicon; Johnson & Johnson, São José dos Campos, SP, Brazil).

### 2.10. Histological Assessment

**Figure 1:**
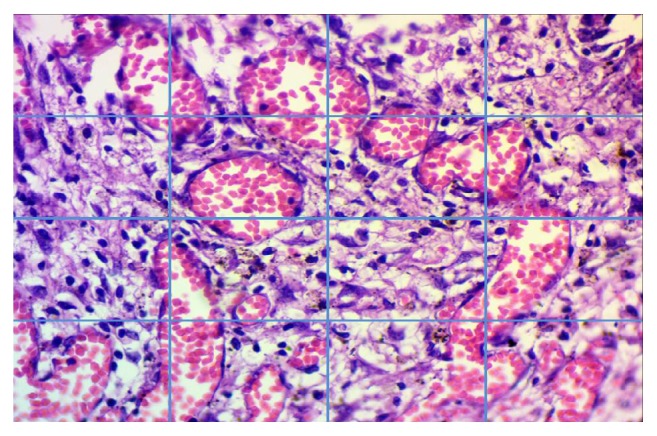
Designing a method for inflammatory cells counting (H&E stains, 400 X).

At 1, 4, and 8 weeks, the rats were sacrificed and the cutaneous incised wounds were separated. All skin tissue specimens were fixed in 10% neutral-buffered formalin for at least 24 hrs, at room temperature. After fixation, the skin sections were dehydrated in graded ethanol, cleared in xylene, and embedded in paraffin. Tissue slices of 4 *μ*m thickness were attained and stained using the standard H and E technique, mounted on glass slides, and visualized by light microscopy. Histopathological examinations were performed in double-blind fashion, under a microscope from 40X to 400X magnifications. Inflammatory cells were quantitatively assessed in the site of the opening tube in the subcutaneous tissue that contained treated product or was empty in the case of the control group, in 2 arbitrarily chosen fields at 400X utilizing a light microscope (Leica Motic), connected with an image analyzer software (ToupTek, ToupView, 86X, 3.7.4183, 2014). For each area, a picture was captured and then partitioned into 16 squares (Figure 1), after which all of the inflammatory cells (polymorphonuclear cells and mononuclear cells) were counted and an average number for each group was achieved. Additionally, the counted inflammatory cells were calculated in each group for the three study durations. The inflammatory cells were scored and classified as follows: negative reaction or score 0 (0-25 inflammatory cells), mild or score 1 (26-50 inflammatory cells), moderate or 2 score (51-75 inflammatory cells), and severe or 3 score (more than 75 inflammatory cells).

### 2.11. Statistical Analysis

A statistical software package, SPSS (version 22.0, Chicago IL, USA), was used to perform statistical analysis. The statistical analysis of variation among the experimental groups was performed by One-way ANOVA, and in order to make a pairwise comparison between groups, we used Tukey's Test and also the Paired T test for the dependent variables to obtain separate averages for the polymorphonuclear and mononuclear inflammatory cells among the groups. Values were presented as mean ± standard deviations (SD) and p < 0.05 was regarded as statistically significant.

## 3. Results

Table 1 shows results obtained by GC-MS analyses of the essential oil of* P. atlantica Kurdica* gum with their retention times, retention indices, and percentage shares, in which twenty-nine compounds were identified and alpha-Pinene (79.76%) has been reported as the main compound from the* Pistacia atlantica Kurdica *gum essential oil.

**Figure 2:**
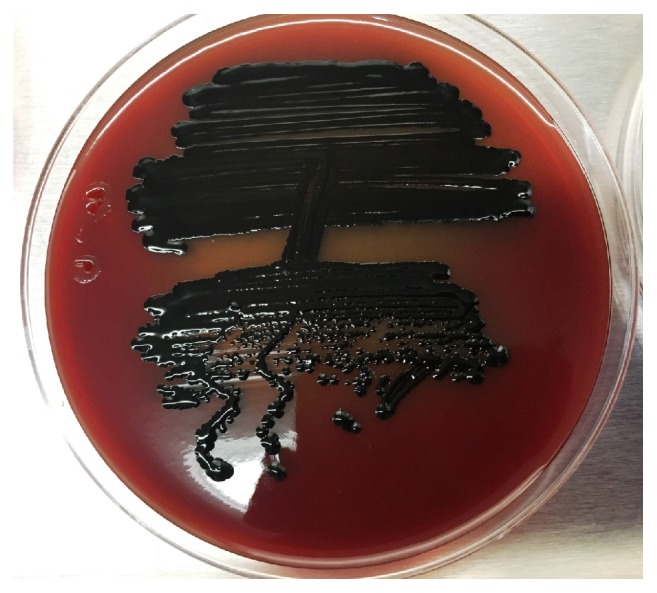
*P. gingivalis* on blood agar.

**Figure 3:**
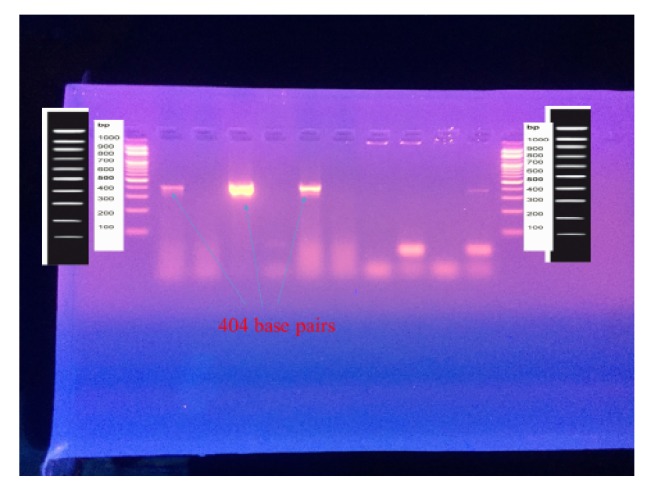
Agarose gel electrophoresis of PCR product for* P. gingivalis*.

The* P. gingivalis* used in this study to determine the MIC and MBC of the extract was clinically isolated from subgingival plaque from the deepest periodontal pockets, the bacteria identification done by colony morphology in which the colony appeared on the plate after 48 hours as small, round, opaque, and convex with black pigmentation developed after one week as shown in Figure 2 after doing gram staining, biochemical identification by VITEK 2 (ANC kit from biomériex) probability 97%, and then bacterial identification confirmed by PCR technique and sequencing, which was 100% similar with ATCC 33277 strain (sequence ID:NC 010729).  Figure 3 shows the three bands of template size of* P. gingivalis*.

The MIC of the essential oils of the gum of* P. Atlantica* against* P. gingivalis* in this study was 12.5*μ*L/ml and the MIC was equivalent to the MBC of the oil against the* P. gingivalis* which was 12.5*μ*L/ml.

**Figure 4:**
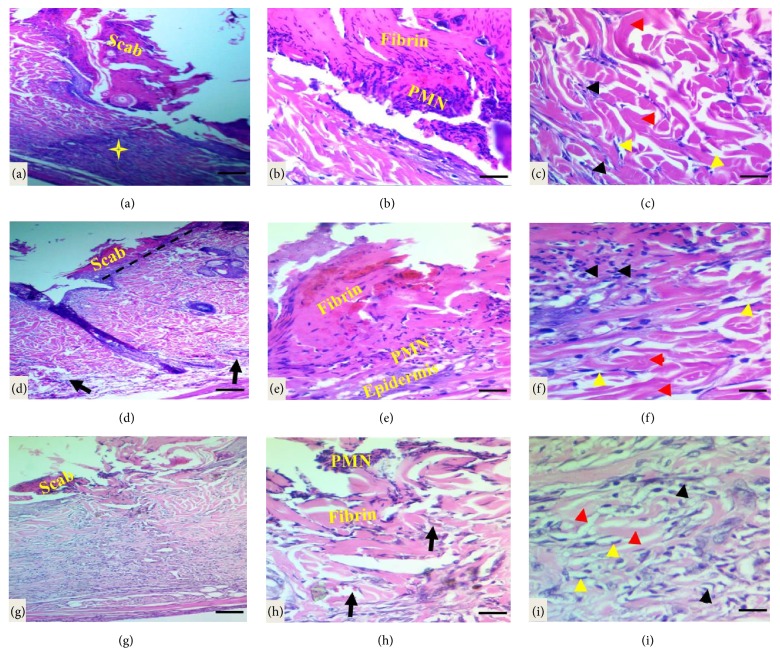
Light micrograph sections of cutaneous wound site at one week after wounding in different groups. (a)–(c) In the control group the wound did not close completely and the wound surface covered with scab and necrotic debris, marked infiltration of neutrophils (yellow star and black arrows) in the dermis with loss of matrix and disorganized collagen fibers (red head arrows, the proliferation of fibroblast as indicated by yellow head arrows). (d)–(f) Light micrograph sections in the tetracycline-treated group showed clear line of demarcation (black dash line); the fibrin and PMN cells bridged the whole incision, moderate infiltration of inflammatory cells in dermis and hypodermis (black arrows) with the proliferation of fibroblast and newly unorganized collagen formation. (g)–(i) In the* Pistacia atlantica Kurdica *gum oil treated group the incised wound closed completely; the fibrin and PMN cells bridged the whole incision with necrotic debris in the surface, moderate infiltration of inflammatory cells in dermis and hypodermis, with angiogenesis as indicated by black arrows with the proliferation of fibroblast (yellow head arrows) and new collagen formation as indicated by black arrows (H&E stain, scale bar 100 *μ*m, scale bar 50 *μ*m, and scale bar 20 *μ*m).

*Histopathological Evaluations of Cutaneous Incised Wound Healing*. The histological evaluation of healed wound area at 1 week after wounding revealed granulation phase or inflammatory phase, for instance, the cutaneous wound, did not close completely and showed a scab formed by necrotic tissue remnants, fibrin, and polymorphonuclear cells infiltration with marked inflammatory cell infiltration in dermis and hypodermis in control group. Additionally, collagen fibers were seen in disorganized arrangement with fibroblast proliferation seen in the dermal layer (Figures 4(a)–4(c)). A clear line of demarcation seen between scab and dermis under the necrotic tissue in a tetracycline-treated group in which the wound closed if compared to the control group, moderate infiltration of inflammatory cells in epidermis extends to dermis and hypodermis with the presence of newly formed collagen fibers and fibroblast proliferation (Figures 4(d)–4(f)). While in* Pistacia atlantica Kurdica *gum oil treated group the demarcation line was completely clear, and the wound more progressed in healing if compared to the control and tetracycline groups, the fibrin net was rich in PMNs. The regeneration of the epidermis was completely started; moderate infiltration of inflammatory cells, the proliferation, and migration of fibroblasts were obvious. Angiogenesis was observed, but the number of fibroblasts increased in the dermis near the wounded area with new collagen formation (Figures 4(g)–4(i)).

**Figure 5:**
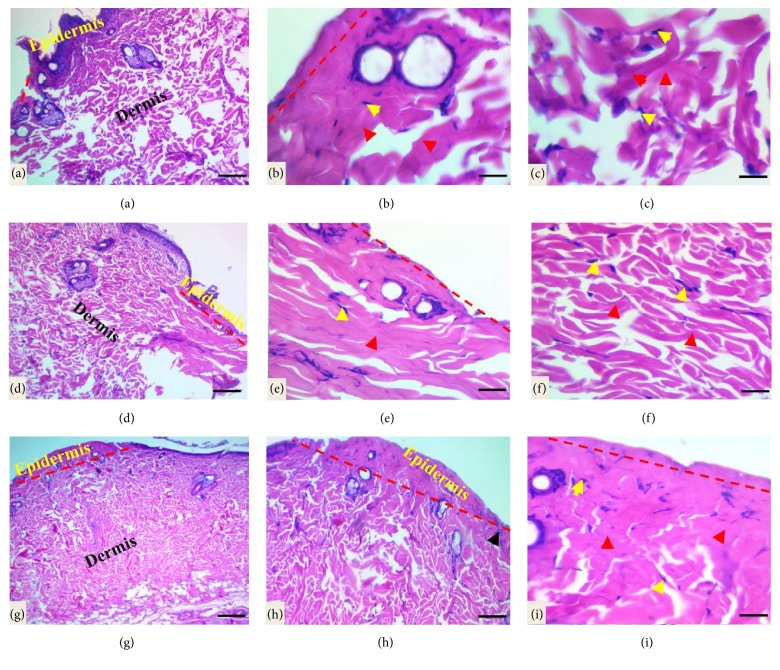
Light micrograph sections of the cutaneous wound site at one month after wounding. (a)–(c) Early reepithelization by forming immature disorganized epidermis and completely closed the gap (red dash line), increased in thickness of dermal layer by of individual and bundle of collagen fibers (red head arrows) with proliferative fibroblasts as indicated by yellow arrows in the control group. (d)–(f) Typical hyperplastic-immature epidermis as indicated by red dash lines with increased in thickness of the dermal layer by intense, well arranged mature bundle of collagen fibers with proliferative fibroblasts in the tetracycline-treated group. (g)–(i) In the* Pistacia atlantica Kurdica *gum oil group the wound healing showed early reepithelization by forming immature disorganized epidermis (red dash line), increased in thickness of the dermal layer by great, mature bundle of collagen fibers with proliferative fibroblasts (H&E stain, scale bar 100 *μ*m, scale bar 50 *μ*m, and scale bar 20 *μ*m).

At 4 weeks after wounding, more progress in wound healing (proliferative phase) was apparent; in the control group an early phase of reepithelization process was seen; the immature-hyperplastic and disorganized epidermis superimposing the area of the wound increased in thickness of dermal layer by deposition of unorganized collagen fibers as a bundles or individual with fibroblast proliferation (Figures 5(a)–5(c)). While late phase of the reepithelization process was seen in treated groups, for example, reepithelialization was markedly completed and tissue regeneration had better quality, collagen fibers were thicker and denser in the tetracycline-treated group (Figures 5(d)–5(f)). Whereas in* Pistacia atlantica Kurdica* gum oil treated group the healing became more evident and the epidermis showed a typical histological picture of the proliferative phase, immature-hyperplastic and disorganized epidermis overlying the area of the wound, at the layer of dermis, fibroblasts were vertically oriented predominantly, and mature collagen fibers exhibited more evident organization pattern than the tetracycline-treated group also control group (Figures 5(g)–5(i)).

**Figure 6:**
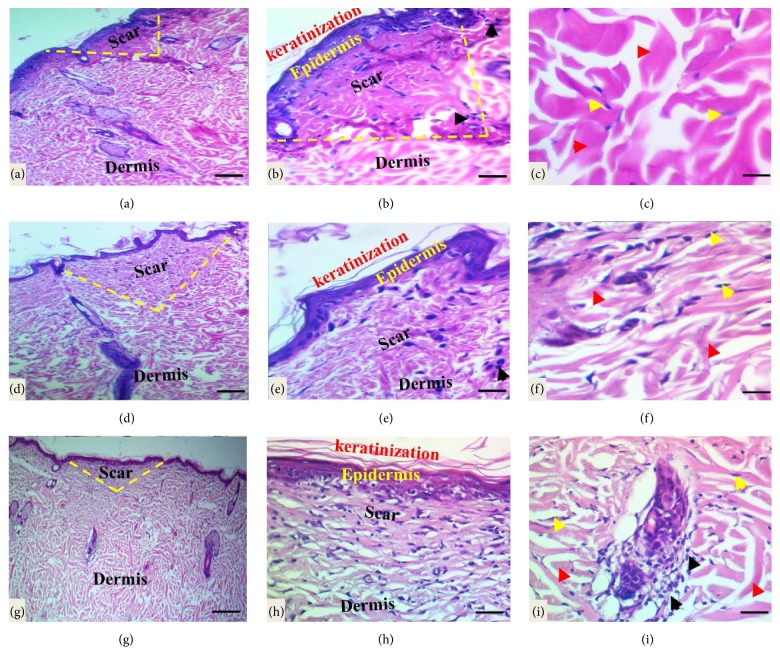
Light micrograph sections of the cutaneous wound section at two months subsequently wounding. (a)–(c) In the control group the section showed the new uneven epidermis formed with a minimum keratinization thickness, moderate-marked density of scar tissue (yellow dash lines) in center of the wound with infiltration of inflammatory cells (black head arrows), and well-organized thick bundles of collagen fibers (red head arrows) with normal fibroblast morphology as indicated by yellow arrows. (d)–(f) Well-organized epidermis with mild keratinization, mild-moderate scar intensity (yellow dash lines) with minimum inflammatory cells infiltration and starting the appearance of rete ridges or dermal papillae, and well-organized thick bundles of collagen fibers with normal fibroblast features in the tetracycline-treated group. (g)–(i) The wound in* Pistacia atlantica Kurdica *gum oil group is closed with a thick-uniform new epidermis with the full thickness of keratinization. The scar tissue (yellow dash lines) at the center of wound is mild-moderate in density laden with mild inflammatory cells infiltration (red arrows); compact and regularly arranged collagen fibers (black head arrows) observed in dermis with normal fibroblast as indicated by yellow head arrows (H&E stain, scale bar 100 *μ*m, scale bar 50 *μ*m, and scale bar 20 *μ*m).

Histological analysis of healed wound area in the control group at 8 weeks after wounding revealed early remodeling phase, wounds are fully reepithelialized with minimum keratinization, scar tissue formed with marked intensity and loaded with the mild infiltration of inflammatory cells (neutrophils, macrophages, lymphocytes, and fibroblasts), and well-organized thick bundles of collagen fibers were also observed (Figures 6(a)–6(c)), while in treated groups the wound showed late stage of remodeling phase with full maturation of the wound, and the final steps of dermal reorganization are proceeding but with the variable levels; for instance, in tetracycline-treated group, the mature wound showed well-organized epidermis with dermal papillae development, while the scar intensity ranged from mild-moderate and minimum degree of inflammatory cells infiltration and well-organized thick bundles of collagen fibers (Figures 6(d)–6(f)), in comparison to the* Pistacia atlantica Kurdica *gum oil treated group (Figures 6(g)–6(i)); reepithelialization was markedly completed and tissue regeneration had better quality, the mature wound revealed the remodeling phase including normal thickness of epidermis and keratinization and mild-moderate scar tissue intensity with mild numbers of inflammatory cells with well-organized thick bundles of collagen fibers in the dermal layer, but the cutaneous annexes do not appear.

*Assessment of the Inflammatory Reaction or Process in the Opening Tube Site*. The statistical analysis of polymorphonuclear and mononuclear inflammatory cells at three different durations among variable groups was showed in Table 2. In control group the raising of inflammatory reaction surrounding the open tube by increasing the mean numbers and scores of polymorphonuclear inflammatory with scores 1 and 0 for neutrophil (4.800±2.65) and eosinophil (2.00±1.15), respectively, was demonstrated, but the differences were significant only for eosinophil; mononuclear inflammatory cells showed a highly significant with scores 3, 0, and 3 for the lymphocytes, plasma, and macrophages cells in the first week, whereas the mean number of the inflammatory cells in the tetracycline-treated group was maintained on the same level with exception of eosinophil increased to 4.20±3.55, and the plasma cells slightly decreased (1.50±1.26), while the scores for the each cells were 1 and 1 for the polymorphonuclear cell and 3, 0, and 3 for the mononuclear inflammatory cells that showed significant effect. In the* P. atlantica kurdica* oil treated group the inflammatory cells significantly increased in comparison to the control and tetracycline-treated groups that showed significant values with scores of 1 and 3 for the neutrophils and eosinophils and also 3, 0, and 3 for the mononuclear inflammatory cells with exception of the plasma cells (0.80±0.91) and macrophages (10.50±4.85) were dropped.

Additionally, the inflammatory reaction in 4 weeks significantly regressed in the all three groups in comparison to the first week. The reduction of the inflammatory cells particularly the polymorphonuclear was observed in the control group with scores including 0 and 1 that revealed a significant decreasing particularly in the neutrophil cells, while the mononuclear inflammatory cells remain stable and scored as 3, 0, and 3, whereas significant decreasing was shown in the tetracycline group in comparison to the control group particularly the number of neutrophil (0.00±0.00) in which the major marker of acute inflammation and additionally the scores also reduced to the 0, 0, 3, 0, and 1 for the polymorphonuclear and mononuclear inflammatory cells, respectively, with exception of lymphocytes being not decreased. Furthermore, the polymorphonuclear inflammatory cells in the* Pistacia atlantica Kurdica *gum oil treated group were slightly increased with scores of 0 and 1 in comparison to the tetracycline groups particularly eosinophil, while the mononuclear inflammatory cells significantly decreased in comparison to the both groups with scores of 3, 0, and 1 and more specifically control group.

The inflammatory process at the 8 weeks of this study expressively was decreased in the all groups if compared to the first week and showed that the both types of inflammatory cells were decreased in the tetracycline, where scored as 0, 0, 0, 0, and 1, while the* Pistacia atlantica Kurdica *gum oil treated groups scored as 0, 3, 1, 1, and 0 in comparison to the control group where found scores of 0, 0, 3, 0, and 1 for the polymorphonuclear and mononuclear inflammatory cells correspondingly, with exception of eosinophil increased significantly in the* P. atlantica* oil treated group (9.40±6.50) and scored 3, whereas inflammatory cells in the* P. atlantica* oil treated group were increased slightly in comparison to the tetracycline group with exception of the macrophages which were declined (2.20±1.54).

## 4. Discussion

The gum exudates from species of the* Pistacia *genus have been used for treatment of gastrointestinal disorders over a long period, at least ten centuries [20]; the antimicrobial effect of the essential oils of mastic gum against a number of microorganisms has been shown* in vitro* such as* Staphylococcus aureus*,* Klebsiella pneumoniae, Escherichia coli, Candida albicans *[21],* E. coli*,* S. aureus, *and* S. pyogenes *[16].

This study represents the first reported investigation into the composition and antibacterial effect of essential oil of* Pistacia atlantica Kurdica* gum in Kurdistan, Iraq, against clinically isolated periopathogenic bacteria (*P. gingivalis*).

GC-MS analyses of the essential oil of* Pistacia atlantica Kurdica *gum revealed that 79.76% of essential oil of* Pistacia atlantica Kurdica *gum grown in Kurdistan, Iraq, was alpha-Pinene and this finding is consistent with [10, 17, 22, 23]; in their studies it was reported that alpha-pinene was 97.18%, 92.42% in male trees, 84.10% in female trees, 81.9%, and 91.47%, respectively; these variations in percentage amount of pinene might be due to the difference of harvesting time, climatic conditions, geographical origin, and sex of cultivars and these differences in the chemical compositions of essential oils are responsible for their medicinal effects or their biological activity which varies from one area to another [24, 25]. The MIC of the essential oils of the gum of* Pistacia atlantica Kurdica *against* P. gingivalis* in this study was 12.5*μ*L/ml by using serial dilution method and the MIC was equivalent to the MBC of the oil against the* P. gingivalis*, indicating a bactericidal effect of the oil. The difference between bactericidal or bacteriostatic effect is clinically important in that bactericidal agents may be more effective in the treatment of diseases [22].

As Memariani et al. [23] in their study stated that alpha-pinene at high concentration causes disruption of bacterial cell membrane integrity; this function is the reason for its bactericidal activity.

Histological process in wound healing is three stages including inflammation, proliferation, and remolding [26]. Wound healing is a dynamic and complex process in which the damaged tissue layers and cellular structure should be restored into the normal state as closely as possible. Lipid peroxidation is an important process wound healing. Collagen fibrils viability increases by inhibiting lipid peroxidation [27].

Histological findings of this study on 1 week showed that the tissue regeneration is much better in skin wounds treated with tetracycline and* Pistacia atlantica Kurdica* gum oil than in control skin wounds. Although these results were based on the profitable effects of the both treated materials on the morphology of dermal wound healing, yet the newly synthetized collagens were still unorganized and distributed randomly in all rats at this stage. In the current study better healing progression in rat of* Pistacia atlantica Kurdica *gum oil treated group due to the presence of phenols and flavonoids that reduce the lipid peroxidation and can improve vascularity, collagen synthesis promotes cross linking of collagen because the Pistacia phenolics act as primary antioxidants or free radical scavengers and this finding in accordance with the previous studies that evaluated the histological examination of wound healing and indicated that the phenolics component of the* Pistacia atlantica Kurdica *can reduce damaged tissue layers and accelerate wound healing [28].

In our result the mean and score of inflammatory cells increased significantly in* Pistacia atlantica Kurdica *gum oil treated group than the control and tetracycline groups, particularly the neutrophil and the lymphocytes. This is the one positive effect of* Pistacia atlantica Kurdica gum oil* in enhancing capacity of wound healing by increasing the immunity via activation and recruitment of neutrophils, also increasing effectiveness in mediating a granulation tissue response which is important in this period of wound healing [29].

In current study on 4 weeks, the* Pistacia atlantica Kurdica *gum oil treatment and tetracycline treatment groups had the same effect and showed the presence of full thickness epidermal layer which covered completely the wound area and showed a proper reepithelialization with decreasing in cellularity and more organization of the collagen fiber orientation. These results in agreement with studies that showed* Pistacia atlantica Kurdica *gum oil as gels improved epithelialization through better organization of collagen fibers [30] or due to the anti-inflammatory activity of this extract through regression of mononuclear inflammatory cells with enhancing the fibroblast growth and proliferation leading to fastening of the healing [31].

The mean numbers and the scores of the inflammatory reaction that surrounded opening tube were regressed in the all groups significantly, even within the same group when compared to the 1 week of the study, particularly the mononuclear inflammatory cells in the* Pistacia atlantica Kurdica *gum oil treated groups if compared to the tetracycline drug while the polymorph nuclear cells slightly increased; this may be the irritant influence of high contents of extracts during the wound healing process as mentioned before in the stages of wound healing [32] and also for increasing the eosinophil throughout the study in this group.

The results of this study demonstrated that the wound healing and repair in the 8 weeks were more evident and had better remodeling in the treated groups than the control group, particularly in the rats of the* Pistacia atlantica Kurdica *oil treated group, that showed complete reepithelialization and tissue regeneration but the cutaneous annexes do not appear in comparison to the tetracycline-treated group. Moreover, reduction in total cellularity and enhancing fibroblast maturation and differentiation were observed in the wound area in both treated group that in accordance with the study of Kahkeshani et al. said that a plant to be an effective wound healer, its active constituents need to have anti-inflammatory, antimicrobial, and antioxidant activities. These are the important biological activities that are paid attention to for development of new products for wound healing [33].

Additionally, the inflammatory cells dropped in the all groups in comparison to the first week more specially in the* Pistacia atlantica* oil treated groups that confirmed by significant values and scores 0, 3, 1, 1, and 0 in comparison to the tetracycline group where found scores of 0, 0, 0, 0, and 1 for the polymorph nuclear and mononuclear inflammatory cells correspondingly, and it is an interesting founding for such a product which had anti-inflammatory effects related to the oleanonic acid in its composition that reduced the production of leukotriene B4 [34], and our product was considered as biocompatible because the inflammatory reaction severity was decreased by times surrounding the opening tube [35, 36]

## 5. Conclusion

For the first time, the antibacterial activity of essential oil of gum of* Pistacia atlantica Kurdica *in Kurdistan region, Iraq, against clinically isolated periopathogen* p. gingivalis in vitro *model is reported. The study revealed that the extract had a significant antibacterial effect. The polyphenols and flavonoides components of the gum extract oil are a promising source in wound healing as a powerful antioxidant and anti-inflammatory agent. Therefore it can be considered as a natural adjuvant herbal therapy for periodontitis.

## Figures and Tables

**Table 1:** GC analysis of chemical composition of essential oil of *Pistacia atlantica Kurdica. *

Peak#	R.Time	Area	Area%	Name
1	4.641	1594271	0.63	Bicyclo[4.1.0]hept-3-ene, 3,7,7-trimethyl-, (1S)-
2	4.938	201094121	79.76	alpha.-Pinene
3	5.034	6478085	2.57	Bicyclo[2.2.1]heptane, 2,2-dimethyl-3-methylene-, (1S)-
4	5.292	3312096	1.31	Bicyclo[3.1.0]hexane, 4-methylene-1-(1-methyl ethyl)-
5	5.357	11632007	4.61	Bicyclo[3.1.1]heptane, 6,6-dimethyl-2-methylene-, (1S)-
6	5.490	2708744	1.07	beta-Myrcene
7	5.613	402474	0.16	1,5,5-Trimethyl-6-methylene-cyclohexene
8	5.658	290088	0.12	Guanosine, 2'-deoxy-N-(trifluoroacetyl)-, 3',5'-bis(trifluoroacetate)
9	5.727	1832318	0.73	3-Carene
10	5.821	315065	0.12	(+)-4-Carene
11	5.914	1013121	0.40	Benzene, 1-methyl-3-(1-methyl ethyl)-
12	5.973	5376458	2.13	D-Limonene
13	6.309	177519	0.07	1,4-Cyclohexadiene, 1-methyl-4-(1-methyl ethyl)-
14	6.626	2935735	1.16	(+)-4-Carene
15	6.772	220829	0.09	1,6-Octavian-3-ol, 3,7-dimethyl-
16	7.081	506276	0.20	3-Cyclopentene-1-acetaldehyde, 2,2,3-trimethyl-
17	7.256	856329	0.34	Bicyclo[3.1.1]heptan-3-ol, 6,6-dimethyl-2-methylene-, [1S-(1.alpha.,3.alpha.,5.alpha.)
18	7.301	1136279	0.45	Bicyclo[3.1.1]hept-3-en-2-ol, 4,6,6-trimethyl-
19	7.392	26	0.00	3-Cyclohexene-1-methanol, 2-hydroxy-.alpha.,.alpha.,4-trimethyl-
20	7.568	234560	0.09	Cyclohexene, 3-acetoxy-4-(1-hydroxy-1-methyl ethyl)-1-methyl-
21	7.660	232216	0.09	3-Cyclohexene-1-ol, 4-methyl-1-(1-methyl ethyl)-, (R)-
22	7.737	439885	0.17	Thymol
23	7.825	2949602	1.17	Decane, 3,7-dimethyl-
24	8.684	427058	0.17	Acetic acid, 1,7,7-trimethyl-bicyclo[2.2.1]hept-2-yl ester
25	8.844	2109901	0.84	Phenol, 2-methyl-5-(1-methyl ethyl)-
26	9.000	200523	0.08	1-Cyclopentene-1-methanol, 2-methyl-5-(1-methyl ethyl)-
27	9.714	2620105	1.04	Tetradecane
28	11.381	792959	0.31	Eicosane
29	18.748	235099	0.09	Phthalic acid, bis(7-methyl octyl) ester
		252123749	100.00	

**Table 2:** The mean ± SE of the inflammatory cells in the all three groups.

Days and Variables	Mean ± SD		
	Control	Tetracycline treatment group	P. atlantica oil treatment group
	(n=15)	(n=15)	(n=15)
*One week*			
Neutrophil	4.800±2.65	3.00±3.01	3.900±1.59
Eosinophils	2.00±1.15*∗∗*	4.20±3.55	7.70±3.83*∗∗*
Lymphocytes	22.80±7.82*∗*	18.00±14.86	41.80±23.48*∗*
Plasma cells	1.60±0.84*∗*	1.50±1.26	0.80±0.91*∗*
Macrophages	22.50±10.44*∗∗*	12.40±5.70*∗*	10.50±4.85*∗∗*
*Four weeks*			
Neutrophil	1.80±1.39^*∗*^	0.00±0.00^*∗*^	2.50±2.46
Eosinophils	2.90±2.07	1.90±0.99	4.70±6.10
Lymphocytes	21.50±12.03^*∗*^	19.70±11.33	10.90±6.10^*∗*^
Plasma cells	1.60±1.34^*∗*^	1.10±0.99	0.80±0.78^*∗*^
Macrophages	13.70±6.20^*∗∗*^	12.20±1.73^*∗∗*^	3.90±1.59^*∗∗*^
*Eight weeks*			
Neutrophil	1.50±1.84	0.10±0.31	0.40±0.51
Eosinophils	1.40±0.69^*∗∗*^	2.30±2.49	9.40±6.50^*∗∗*^
Lymphocytes	10.00±3.39^*∗∗*^	7.01±2.02^*∗∗*^	8.20±3.42^*∗*^
Plasma cells	0.70±1.05^*∗*^	0.10±0.31	0.30±0.67^*∗*^
Macrophages	4.10±1.52^*∗∗*^	3.50±3.02	2.20±1.54^*∗∗*^

The average of inflammatory cells is expressed by mean ± standard error in the same row with different superscripts which differ significantly, *∗*p≥0.001; *∗∗*p<0001 vs. control.

## Data Availability

The data used to support the findings of this study are available from the corresponding author upon request.
